# When adherence to CPAP fails, how do we treat workers with obstructive sleep apnea?

**DOI:** 10.5935/1984-0063.20220012

**Published:** 2022

**Authors:** Maria de Lourdes Rabelo Guimarães, Pedro Guimarães Azevedo, Sérgio Barros-Vieira, Maxime Elbaz, Damien Leger, Ana Paula Hermont

**Affiliations:** 1 Universidade Federal de Minas Gerais, Faculty of Medicine, Belo Horizonte, Brazil, - Belo Horizonte - Minas Gerais - Brazil.; 2 UNISONO- Centre de Diagnostique et Traitement des Troubles du Sommeil, - Vitória - Espírito Santo - Brazil.; 3 Université Paris Descartes, Sorbonne Paris Cité, EA, Vigilance Fatigue Sommeil et Santé Publique - Paris - Paris - France.; 4 Hôtel-Dieu, centre du Sommeil et de la Vigilance, Consultation de pathologie professionnelle Sommeil Vigilance et Travail, - Paris - Paris - France.; 5 Universidade Federal de Minas Gerais, Faculty of Dentistry, Belo Horizonte, Brazil, - Belo Horizonte - Minas Gerais - Brazil.

**Keywords:** Oral Appliances, Mandibular Advancement Device, Obstructive Sleep Apnea, Occupational Medicine, Prevention Of Accidents

## Abstract

**Aims:**

A cross-sectional study was designed to evaluate the effectiveness of a mandibular advancement device (MAD) with respect to respiratory and sleep parameters among miners with obstructive sleep apnea syndrome (OSAS) and primary snore.

**Methods:**

The target sample was composed by 102 Brazilian miners with a history of non-adherence to continuous positive airway pressure. All patients were treated with a MAD and underwent pre and post-treatment full-night polysomnography. Ethical approval and consents were obtained. Bivariate and logistic regression analyses were conducted. The level of statistical significance was set at 5%.

**Results:**

After the treatment with MAD, 71.8% of patients presented a decrease ≥ 50% in the basal apnea-hypopnea index (AHI), 51.2% presented an AHI < 5 events/h and 83.3% reached an AHI<10/h, whereas 22.5% did not show any changes and 7.5% of the sample presented an increase in the AHI (p<0.05). There was an increase in the mean SpO2 nadir (p<0.001) and in the baseline duration of the REM sleep stage (p<0.05). The MAD significantly decreased snore events (p<0.05). Multivariate analysis did not identify predictive factors related to therapy success (decrease ≥ 50% of AHI). However, basal AHI was a significant predictor related to the secondary endpoint (AHI<10/h) (OR= 1.06, IC 95%1.00-1.13, p=0.007).

**Conclusions:**

The MAD therapy showed significant improvements in AHI, minimum oxygen saturation, REM sleep and snoring.

## INTRODUCTION

Mining has always been among the most dangerous occupations, and with the growing demand for minerals, mine safety assumes even greater importance^1^. Traumatic injuries in mining remain a significant problem ranging from trivial to fatal injuries^2^. Sleep appears to be a much easier way to determine if a worker is tired, and fatigue presents several challenges for the mining industry^3^. Miners may have poorer sleep quality and less sleep efficiency compared to the general population of the same age, with nearly 40 to 60 minutes less bed time and total sleep time before starting the day shift^4^.

Obstructive Sleep Apnea Syndrome (OSAS) is characterized by continuous collapses of the superior airway during sleep, associated with nocturnal hypoxemia and sleep fragmentation^5^. Brain dysfunctions might also manifest, such as excessive daytime sleepiness and lack of concentration. These are the most common causes of traffic accidents^6^. OSAS compromises professional activities leading workers to higher risks of occupational accidents^7,8^, increasing the incidence of illness and decreasing worker’s productivity^9^.

In North America, OSAS’s prevalence varies from 20 to 30 % in men and 10 to 15 % in women when it is defined as an apnea-hypopnea index (AHI) higher than five events per hour^10^. In Canada, the evaluation of mine workers subjected to polysomnographic exams showed that the most fatigued individuals presented significantly more sleep pathologies than less fatigued workers^11^. OSAS was the most prevalent sleep disorder in the fatigued workers^12^. In Turkey, a statistically significant correlation between Epworth Sleepiness Scale (ESS) and work-related accidents has been found. However, the prevalence of OSAS symptoms in miners was similar to the general population^13^. In Brazil, a study conducted in 2010 showed an OSAS’s prevalence of 32% in general population and 30.1% in workers^14^.

OSAS treatment could decrease the number of workrelated accidents and improve work performance^6,8^. Studies have shown that treatment with continuous positive airway pressure (CPAP) devices can markedly reduce the risk of accidents^15^, however, there is a lack of studies evaluating the effectiveness of oral appliances (OA) in occupational field.

CPAP is considered the first-line treatment for patients with moderate or severe OSAS associated with somnolence^16^. Nevertheless, the adherence rate to CPAP is problematic^17^, since 46 to 83% of patients do not use CPAP for more than four hours a day, leading to the need of alternative treatments^18,19^. OA have emerged as an alternative to CPAP therapy, the most commonly used OA reduce upper airway collapses by advancing the mandible and they are known as mandibular advancement devices (MAD)^20^. OA improve subjective somnolence and the rates of sleep respiratory disturbances in patients with OSAS^9,21^. Studies comparing both therapies have shown that MAD present effects similar to the gold-standard CPAP treatment in improving cognitive development and decreasing excessive daytime sleepiness^9,22^.

Moreover, patients often prefer MAD to CPAP treatment, and the superiority of CPAP in reducing OSAS parameters on polysomnography does not necessarily result in better health outcomes in clinical practice. Comparable effectiveness of MAD and CPAP has been attributed to higher MAD compliance. Therefore, despite being less effective in reducing apneic events, MAD may be counteracted by greater treatment adherence^23^. The aim of this study was to evaluate the effectiveness of a MAD with respect to respiratory and sleep parameters among Brazilian miners with obstructive sleep apnea. Predictors of success were also evaluated.

## MATERIAL AND METHODS

### Ethical issues

The study was conducted according to the principles of the Helsinki Declaration and received the approval of the Internal Review Board of Hospital Felício Rocho BH-MG (CAAE – 0002.0.240.000-11). Patients’ consents were obtained from the entire sample.

### Participants

The present sample was composed by 102 miners suffering from primary snoring and/or OSAS who presented a history of non-adherence to CPAP. Those miners were referred by sleep specialists for dental clinic specialized in the treatment of OSAS with MAD. Data collection occurred between October of 2016 and December of 2017. All miners who were referred to the dental clinic during this period and attended the eligibility criteria (n=102) were invited to take part in the study.

### Eligibility criteria

The including criteria were: age ≥ 18 years-old; primary snoring or OSAS (AHI >5/h) during polysomnography (PSG); refusal or non-adherence to CPAP (due to pressure intolerance, skin irritation, mask leak with secondary sleep disturbance, claustrophobia, aerophagia, compliance < 3h/night). All patients underwent detailed clinical examination to evaluate whether they fit the primary or secondary indications for the therapy with MAD.

The exclusion criteria were defined as: central apnea index ≥ 5/h, severe sleep comorbidities other than OSAS or coexistent psychiatric diseases, patients with insufficient number of teeth for fitting the MAD, active periodontitis and acute temporomandibular disorders (TMD).

### Polysomnography

All patients were submitted to two full-night polysomnography recordings (basal and after-treatment). All exams were conducted at the same sleep laboratory under the supervision of a capacitated technician and the reports were written by Sleep Medicine physicians and the exams were conducted accordingly to the American Academy of Sleep Medicine^24^.

Obstructive apnea was defined as a pause ≥10 seconds of the air in the nasal cannula and the thermistor. Hypopnea was defined as a decrease in airflow of ≥30% (by a valid measure of airflow) lasting ≥10 s, associated with either ≥3% desaturation from the pre-event baseline or an arousal. The AHI was defined as the number of apneas plus hypopneas per hour of sleep.

The exams with the MAD *in situ* were performed at least three months after the OA was fitted and after it had been optimally titrated. When the therapy did not present response rates (a decrease in the AHI >50% when compared to the basal index and/or persistent symptoms), the MAD was titrated again, and a PSG was performed after a six-month period.

### Intervention: mandibular advancement device

**Figure 1:**
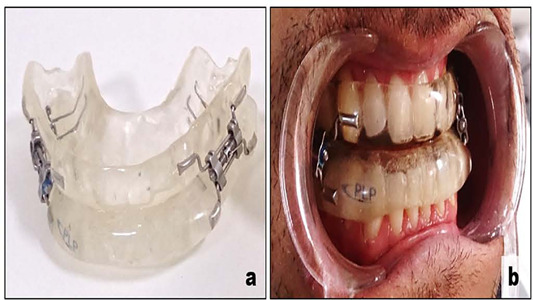
Mandibular advancement device (a); mandibular advancement device in situ (b).

Each patient was treated with a MAD: Lateral- Protrusive Plaque® (PLP) (Figure 1). PLP is a mandibular advancement device developed by one of the authors (MLRG) and consists of two encapsulated acrylic plates retained by the two arcades through retaining clamps providing complete occlusal coverage^25,26^. Maxillary and mandibular plaques are connected by an adjustable screw that allows progressive protrusion of the mandibular component in increments of 0.25mm, comprising a total of 11 mm in anteroposterior movement.

The appliance is fitted with an initial advancement of 50% of the patient maximum mandibular protrusion. The therapy is conducted by qualified dentists and the appliance is titrated according to the dentist criteria, respecting the patient’s tolerability and their reports related to symptoms improvements. After reaching optimal titration (at least 3 months after fitting the MAD), full-night PSG recordings must be performed to check therapy effectiveness.

### Endpoints

The first endpoint was the therapy response rate, which was defined as a decrease ≥ 50% in the AHI. The second endpoint was the evaluation of a complete treatment response defined by an AHI after-treatment: < 5 events/h or < 10 events/h. Patients who achieved response rates were encouraged to continue treatment until they reached a complete response.

### Evaluated parameters

Sleep and respiratory parameters were recorded before the treatment and during the follow-up visits. All patients were referred to the same sleep laboratory for diagnosis and monitoring the therapy with the MAD *in situ*. The independent variables evaluated were: 1) sleep parameters (snore, N3 and REM sleep percentage, arousal index, sleep effectiveness, sleep bruxism); 2) respiratory parameters (AHI, mean and minimum SpO_2_); 3) dental parameters (sleep bruxism and malocclusion); 4) sociodemographic parameters (sex, age, work hours/shift); 5) general health parameters (presence of comorbidities); 6) tolerability, adherence and side effects related to the therapy were evaluated by self-report questionnaires.

### Statistical analysis

Statistical analyses were performed using the SPSS software package (version 25.0 for Windows; SPSS Chicago, IL, USA). Data is expressed as frequency, median and range. Mean and standard deviation were the measures used for data following normal distribution. Basal and after-treatment AHI values were categorized as: 1) Normal (AHI<5 events/h); 2) Mild (AHI = 5-15 events/h); 3) Moderate (AHI = 16-30 events/h); 4) Severe (AHI>30 events/h) and compared using Marginal Homogeneity Test and bivariate analysis (Qui-square test). Mean values for SpO_2_ were compared before and aftertreatment using the t-paired test. A logistic regression analysis was used to determinate the independent factors associated with the effectiveness of the treatment. The level of statistical significance was 5% (p ≤ 0.05).

## RESULTS

### Demographic, anthropometric and baseline clinical parameters

One hundred and two mine workers were referred to the therapy with MAD. Twenty-two (21.6 %) could not be treated with the MAD due to the following reasons: acute TMD, craniofacial dimorphism and compromised periodontal condition. Therefore, 80 patients were included and treated with the MAD. The sample’s mean age was 46.24 ±8.69 years, being 67 (83.8%) men and 13 (16.2%) women. Patients’ sociodemographic data and their baseline clinical profile are shown in Table 1.

**Table 1:** Sociodemographic data, baseline clinical profile and respiratory status of the sample.

Parameters	Baseline data
Sociodemographic data	
Gender (male/ female), n (%)	67 (83.8) / 13 (16.2)
Age (years) (mean ± SD)	46.24 ±8.69
Working Time	
Shift work, n (%)	49 (61.2)
Day shift, n (%)	31 (38.8)
Respiratory and sleep parameters	
AHI* /h (mean ± SD)	16.48 ±13.02
AI**/h (mean ± SD)	1.35 ±1.06
HI***/ /h (mean ± SD)	14.40 ±12.59
SpO2**** (mean ± SD)	94.38 ±0.78
SpO2**** nadir (mean ± SD)	86.33 ±1.53
Arousal index /h (mean ± SD)	20.93 ±11.02
Sleep bruxism, n (%)	21 (26.3)
Angle’s malocclusion	
Class I, n (%)	26 (32.5)
Class II, n (%)	25 (31.3)
Class III, n (%)	29 (36.2)
General health status	
Hypertension, n (%)	14 (17.5)
Diabetes, n (%)	2 (2.5)
Thyroid alterations, n (%)	1 (1.3)
Emotional alterations, n (%)	9 (11.3)
Decrease in libido, n (%)	1 (1.3)
Pulmonary condition, n (%)	4 (5.0)

AHI: apnea-hypopnea index; ** AI: apnea index; *** HI: hypopnea index; **** SpO2: oxygen saturation.

### MAD’s titration

At least 5 ± 2 titrations were required to achieve the optimal MAD titration and the time interval between fitting the OA and the monitoring PSG exam with the MAD *in situ* was 6 ± 3 months. The mean total amount of mandibular advancement was 7.0 ± 1.36 mm.

### Effectiveness

Primary endpoint: 71.8% of patients presented a decrease ≥ 50% in the AHI after-treatment. Secondary endpoint: complete answers to treatment were detected among 83.3% of the patients (AHI<10/h) and 51.2% (AHI <5/h). In summary, after the treatment with the MAD, 70% of the patients presented an improvement in the AHI, 22.5% did not show any changes and 7.5% of the sample presented an increase in the AHI (p ≤0.05).

Table 2 shows the comparison of the sample according to the severity of apnea before and after treatment with the MAD in absolute number and percentage. The parameters adopted with respect to AHI are: normal (patients with AHI < 5events/h), mild (AHI from 5.0 to 14.9/h), moderate (15.0 to 29.9/h) and severe (> 30/h). There was a statistically significant reduction in the AHI after treatment, showing a significant improvement in apnea severity (Table 2).

**Table 2:** Comparison of the sample according to the apnea-hypopnea index (AHI) severity before and after treatment with the mandibular advancement device.

AHI Severity	Baseline n (%)*	After treatment n (%)*	p values**
Normal	8 (10.0)	41 (51.2)	<0.001
Mild	38 (47.5)	32 (40.0)	<0.001
Moderate	21 (26.3)	3 (3.8)	<0.001
Severe	13 (16.2)	4 (5.0)	<0.001
Total	80 (100.0)	80 (100.0)	

*n (%) refers to the number of patients;

**Qui-square test.

A more detailed analysis of patients’ improvement according to apnea severity based on the AHI is presented in Table 3. Among the patients with mild baseline AHI (n=38), 65.7% presented normal AHI after treatment and only 7.8% presented worse values after therapy with MAD. All patients with moderate baseline AHI (n=21) presented an improvement, since 42.9% presented normal AHI and 57.1% presented mild AHI after treatment. Finally, among patients with a severe baseline AHI (n=13), 10 (76.9%) presented a decrease in the index after treatment (Table 3).

**Table 3:** Patients’ distribution according to the severity of apnea-hypopnea index (AHI) before and after treatment with mandibular advancement device.

Baseline AHI n (%)*		Post-treatment			
		Normal n (%)*	Mild n (%)*	Moderate n (%)*	Severe n (%)*
Normal n (%)	8 (100.0)	5 (62.5)	3 (37.5)	0	0
Mild n (%)	38 (100.0)	25 (65.7)	10 (26.3)	2 (5.2)	1 (2.6)
Moderate n (%)	21 (100.0)	9 (42.9)	12 (57.1)	0	0
Severe n (%)	13 (100.0)	2 (15.4)	7 (53.8)	1 (7.7)	3 (23.1)
Total	80 (100.0)	41 (51.3)	32 (40.0)	3 (3.7)	4 (5.0)

*n (%) refers to the number of patients.

### Oxygen saturation and sleep related data

The average of minimum SpO_2_ increased from 84.0 ± 5.9 to 86.9 ± 5.4 (95% CI 1.2-3.5) after treatment (p ≤0.001). There was a significant increase in the baseline duration of the REM sleep stage after the therapy with the MAD (p<0.05). The amount of arousal index per hour decreased independently of the OSAS severity (p<0.001). Changes in baseline sleep effectiveness and in N3 sleep stage were not significant (p>0.05). The MAD therapy decreased snore events (p<0.05).

### Tolerability, adherence and side effects

Adherence to treatment was self-reported and showed that 73.0% of patients used the MAD regularly (>4 days/week, >4h/night) and 27.0% used it irregularly (<4 days/week, <4h/night).

Among the sample, 37.5% reported side effects during the first 6 months of the therapy and 8.8% after 6 months using the MAD. Most of the reported side effects were transitory and presented mild to moderate severity. The most common complaint related to temporomandibular joint (TMJ) pain and alterations in dental occlusion were reported six months after fitting the MAD. Hypersalivation, teeth discomfort, injury to the mucosa and muscle pain were also reported after fitting the appliance, but these side effects were transitory. No patient discontinued the therapy because of side effects during the evaluation period.

### Predictive factors

Multivariate analyses did not identify predictive factors related to the therapy success (decrease ≥ 50% in the AHI). However, basal AHI appeared to be a significant univariate predictor related to the secondary endpoint (AHI<10/h) (OR= 1.06, IC 95%1.00-1.13: p=0.007) (Table 4).

**Table 4:** Predictors of success for mandibular advancement device therapy according to the treatment response rate.

Variables	Response rate (decrease ≥50% in AHI)		OR (95%CI)	p values
	Yes	No		
Sex M/F (%)	58.8 / 11.2	25.0 / 5.0	1.02 (0.25-4.18)	0.973
Malocclusion (%)				
Class I	25.0	7.5	Reference	0.574
Class II	21.3	10.0	0.56 (0.15-2.03)	0.382
Class III	23.8	12.5	1.07 (0.26-4.32)	0.915
Overbite (mm)	3.02 ±2.17	3.10 ±2.08	1.03 (0.78-1.35)	0.807
Overjet (mm)	2.66 ±1.76	2.54 ±1.64	0.94 (0.65-1.35)	0.754
Maximum protrusion (mm)	8.05 ±1.97	8.19 ±1.76	1.00 (0.75-1.34)	0.971
Therapeutically protrusion (mm)	7.46 ±1.47	7.21 ±1.14	0.82 (0.56-1.19)	0.296
Mouth breathing (yes/no %)	27.50 / 42.50	7.5 / 22.50	0.49 (0.15-1.54)	0.226
Oval palate (yes/no %)	66.25 / 3.75	28.75/ 1.25	0.83 (0.06-10.86)	0.893
AHI (mean values)	16.7 ±10.40	16.0 ±19.00	0.99 (0.94-1.04)	0.831
Arousal index (mean values)	12.3 ±8.60	12.5 ±15.00	1.01 (0.94-1.07)	0.931
	Complete response rate (AHI<10/h)			
	Yes	No		
Sex M/F (%)	65.0 / 16.25	18.75 / 0	1.13 (0.98-1.54)	0.056
Malocclusion (%)				
Class I	27.50	5.00	Reference	0.737
Class II	23.75	7.50	1.18 (0.25-5.61)	0.645
Class III	30.00	6.25	2.29(0.46-11.37)	0.440
Overbite (mm)	3.10 ±2.12	2.97 ±2.12	1.03 (0.74-1.41)	0.837
Overjet (mm)	2.63 ±1.80	2.60 ±1.37	0.96 (0.60-1.54)	0.915
Maximum protrusion (mm)	8.13 ±1.88	7.97 ±2.01	0.95 (0.67-1.35)	0.892
Therapeutically protrusion (mm)	7.40 ±1.41	7.30 ±1.24	0.85 (0.53-1.34)	0.837
Mouth breathing (yes/no %)	30.00 / 51.25	13.75 / 5.0	0.52 (0.13-2.04)	0.684
Oval palate (yes/no %)	75.25 / 5.00	18.75 / 0.0	3.92 (0.83-1.97)	0.393
AHI (mean values)	14.60 ±9.90	24.6 ±20.60	1.06 (1.00-1.13)	0.007
Arousal index (mean values)	11.60 ±8.60	15.9 ±16.90	0.97 (0.90-1.05)	0.150

AHI: apnea–hypopnea index; CI: conﬁdence interval; ESS: Epworth Sleepiness Scale; OR: odds ratio.

## DISCUSSION

The present study showed that MAD significantly improved the AHI and minimum SpO_2_, as well as other polysomnographic parameters among the sample. A considerable proportion of shift workers reported sleep symptoms which may be related to the work shift and OSA. A research conducted with shift workers detected that those who reported poor sleep or excessive diurnal somnolence symptoms had their working activities compromised and had four times more risk of suffering work-related accidents ^27^. In addition, a significant relationship has been evidenced between the rates of accidents with professional long-distance drivers with AHI, thus showing that the severity of OSAS is directly proportional to the risk of accidents^28^.

OSA predisposes the patient to several different comorbidities, increasing the need for health services^7,29^. In the present sample, many workers presented comorbidities associated with OSAS, such as hypertension, diabetes, hypothyroidism. OSAS risk assessment in workers and treatment can help reduce the burden of health care on the national system^30^.

Multiple factors can compromise the comparison of data between studies, including the use of different definitions regarding treatment success. The most rigorous definition of success found in studies was AHI ≤ 5/h^21,22^, while the most flexible definition was <50% of baseline AHI^31,32^, as being a success. Comparing our results with data from other researchers^33^, it can be noticed a greater reduction in the AHI in the present sample. This difference may be related to the different designs of MAD used in the study conducted in 2006 in which some participants used titratable appliances and others used monoblocs. In addition, even using the same appliance, different inclusion criteria and different treatment protocols might affect success rates.

The use of OA is currently recommended for the treatment of patients with mild or moderate OSAS (recommendation of level A)^33^. However, in our study, nearly 15.0% of the participants presented severe OSAS (AHI > 30/h) and the MAD was able to significantly reduce AHI. Therefore, while OA can be less effective for the resolution of severe OSAS compared to mild/ moderate cases, patients with severe OSAS that refuse CPAP therapy can be treated with MAD^25,30,34^ ^35^.

The possibility of worsening in respiratory events with the use of OA was reported by other researchers^36,37^. A revision conducted in 1995 showed that 13% of the participants had an increase in AHI after treatment with OA^38^. Our results have shown a worsening among 7.5% of the sample. There are several reasons for the increase in AHI, like the lack of retention of the device in the mouth, invasion of the tongue space or increased vertical dimension caused by the MAD, inadequate degree of protrusion, in addition to factors inherent to the patient, such as non-anatomical phenotype, respiratory diseases, and weight gain during the treatment.

The literature reports that OA provide minimal improvements in minimum oxygen saturation^20,33^. Randomized clinical trials that evaluated 946 adult patients with OSAS treated with OA, detected that the average improvement in oxygen saturation was 3.09%^20^. Our study showed similar results, since the improvement in minimum oxygen saturation varied from 1.2% to 3.5%.

In 2015, it was verified that OA and CPAP do not significantly improve sleep effectiveness and architecture in adult patients with OSAS^20^. Although OA reduce the arousals rates in adult patients with OSAS, CPAP is more efficient^20^. The present study has not found significant N3 sleep improvement, but there was significant improvement in micro arousals and REM sleep rates.

Evaluation of snorers found work-related accidents risks for heavy snorers with apnea and for OSAS patients^39^. In the present study, mine workers with primary snore have been referred to treatment with OA. Oral devices are effective for the treatment of primary snore in adult patients without obstructive sleep apnea^20^. Our results showed that after treatment, there were significant improvements in snore measured during the PSG. Moreover, it is estimated that twice as many chances of accidents are caused by carelessness and somnolence in drivers^40^.

OA therapy was well tolerated among the sample; the side effects were transitory, and no participant discontinued MAD therapy because of side effects during the period of evaluation. The major complaints were pain in the TMJ and alterations in the occlusion. Our results verified that the use of a MAD led to the development of TMD in a small number of patients; but as stated in the literature, these signs are generally transitory^41^. Patients with signs and preexisting symptoms of TMD did not present significant exacerbation of these signs and symptoms with the use of OA^41^.

In the long term, occlusal alterations might occur in up to 85.0% of patients^42^, and these alterations seem to be progressive. In the present study, a short-term evaluation was conducted, and it was observed a decrease in overbite and overjet, besides the opening of diastema in molars.

The prediction of success related to OA therapy is a key-question from both therapeutic and financial perspectives^43^, but the capacity to realize a precise clinical selection of patients for therapy with OA is still limited. Predictive accuracy has shown a broad variability depending on the definitions of success for the treatment^44^. Researchers have found some predictors of success related to treatment with OA such as waist circumference, obesity, overbite, class II division 2 malocclusion^35^. However, in the present study, only baseline AHI proved to be a significant predictor of a complete response rate (AHI <10 / h) during MAD therapy. This difference might rely in the present sample size.

We acknowledge some limitations in the present study, such as the absence of a control group, but our main objective was to evaluate the effectiveness of the MAD during the treatment of mine workers with OSAS. Moreover, adhesion to treatment was based in subjective reports and the results shown are related to short term evaluation. However, as strengths it must be stated that MAD used in the sample (the Lateral-Protrusive Plaque® (PLP)) is an easy-to-adapt progressive mandibular advancement device^26^ that has the particularities of low cost and ease of manufacture. These characteristics facilitate its implementation in a sleep medicine program at companies and other work environments.

## CONCLUSION

MAD therapy is an efficient method to treat mine workers suffering from OSAS and primary snore. The therapy may cause pain in the TMJ and alteration in occlusion, but these effects are often transient and do not lead to treatment discontinuation. The AHI was the only predictor of success related to MAD therapy.
